# Empowering Communities: Culturally Competent Dementia Awareness Education for Older Adults in Miami‐Dade County

**DOI:** 10.1002/alz.093012

**Published:** 2025-01-09

**Authors:** Krisstina Madan, Ellen L Brown, Vanessa Gordon

**Affiliations:** ^1^ Florida International University, Miami, FL USA

## Abstract

**Background:**

The purpose of this Doctor of Nursing Practice Project (DNP) was to explore community stakeholders' perspectives on a newly developed Dementia Awareness Educational Video (DAEV) within a culturally diverse community. Dementia is a global public health concern, with far‐reaching implications affecting millions of individuals, families, and communities. Raising awareness and delivering effective education is paramount, especially within culturally diverse communities.

**Method:**

This qualitative study explored the potential effectiveness of the DAEV. A virtual focus group was conducted. Half (n = 3) of the participants were White and 2 (33%) were Black or African America (one participant was identified as “other”). Most participants were of Hispanic descent (n = 4, 66%). All of participants were highly educated including 2 (33%) participants having a Doctoral degree, 3 (50%) master’s degrees, and 1 (16.7%) bachelor’s degree. A semi‐structured format allowed for exploration of participant perspective on the clarity of the message in the DAEV, overall reaction, and recommendations for community dissemination.

**Result:**

Two main areas of improvement were identified: enhancing message clarity and areas to disseminate the video. To enhance the clarity of the message suggestions included incorporating more images, addressing language accessibility, and ensuring that each video concludes with a clear and concise call to action. Regarding dissemination of the DAEV physician offices, waiting rooms, online platforms, social media and community partners and stakeholder websites were all suggestions.

**Conclusion:**

In the future, these findings will inform the enhancement of the DAEV, ensuring it serves as an effective tool for raising dementia awareness within culturally diverse communities. The collaborative effort among stakeholders, guided by the insights gathered from this focus group, reinforces the commitment to fostering a more dementia‐informed society, where diverse communities receive the support and information, they need to face this critical healthcare issue.

